# The good compliance is an opportunity to avoid pathological brain aging

**DOI:** 10.1192/j.eurpsy.2022.1678

**Published:** 2022-09-01

**Authors:** A. Sidenkova

**Affiliations:** Ural State Medical University, Psychiatry, Yekaterinburg, Russian Federation

**Keywords:** compliance, pathological brain aging

## Abstract

**Introduction:**

Preservation of health, increase in life expectancy determine the need to improve the effectiveness of medical recommendations, which, despite the success of pharmacology, are insufficient for reasons related to the low level of compliance with these recommendations by patients.

**Objectives:**

Participants of the study-148 employees of medical institutions: 12 men, 136 women, their age ranged from 27 to 74 years.

**Methods:**

Despite the absence of signs of decompensation of concomitant pathology, representatives of the subgroups took a different amount of concomitant therapy. Using the scale of assessment of drug compliance, it was found that compliance is most reduced in the subgroup of 41-50 years. In this subgroup, a comprehensive decrease in compliance across the “behavioral”, “emotional”, and “cognitive” domains was detected in 87.8% of cases, while in the younger subgroup partial non-compliance was 32.4%, in the older subgroup - 74.5%

**Results:**

An analysis of the states of cognitive functions in 52 representatives of the middle age subgroup with low compliance rates showed that, unlike other representatives of the same subgroup, their indices for a number of neuropsychological tests are close to the results of more adult participants in the study.Individuals demonstrating low compliance with quite favorable CNS resources are at risk for the formation of pathological aging.

**Conclusions:**

Compliance is considered as Compliance is considered as a control mechanism for preventing normal aging into pathological by regulating risk factors that are dangerous for the brain and associated with the formation of dementia

**Disclosure:**

No significant relationships.

